# Effects of Human Milk Oligosaccharide 2′-Fucosyllactose Ingestion on Weight Loss and Markers of Health

**DOI:** 10.3390/nu16193387

**Published:** 2024-10-05

**Authors:** Joungbo Ko, Choongsung Yoo, Dante Xing, Jisun Chun, Drew E. Gonzalez, Broderick L. Dickerson, Megan Leonard, Victoria Jenkins, Marie van der Merwe, Carolyn M. Slupsky, Ryan Sowinski, Christopher J. Rasmussen, Richard B. Kreider

**Affiliations:** 1 Exercise & Sport Nutrition Lab, Department of Kinesiology and Sports Management, Texas A&M University, College Station, TX 77843, USA; joungboko10@tamu.edu (J.K.); choongsungyoo@tamu.edu (C.Y.); dantexing@tamu.edu (D.X.); chunjs3112@exchange.tamu.edu (J.C.); dg18@tamu.edu (D.E.G.); dickersobl5@email.tamu.edu (B.L.D.); meganleonard10@tamu.edu (M.L.); victoria.jenkins@tamu.edu (V.J.); rjs370@tamu.edu (R.S.); crasmussen@tamu.edu (C.J.R.); 2 Center for Nutraceutical and Dietary Supplement Research, College of Health Sciences, University of Memphis, Memphis, TN 38152, USA; mvndrmrw@memphis.edu; 3 Departments of Nutrition and Food Science and Technology, University of California Davis, Davis, CA 95616, USA; cslupsky@ucdavis.edu

**Keywords:** prebiotic, inflammation, insulin sensitivity, functional capacity, quality of life

## Abstract

Background: 2′-Fucosyllactose (2′-FL) is an oligosaccharide contained in human milk and possesses prebiotic and anti-inflammatory effects, which may alleviate skeletal muscle atrophy under caloric restriction. This study evaluated the impacts of 12 weeks of 2′-FL supplementation in conjunction with exercise (10,000 steps/day, 5 days/week) and energy-reduced (−300 kcals/day) dietary interventions on changes in body composition and health-related biomarkers. Methods: A total of 41 overweight and sedentary female and male participants (38.0 ± 13 years, 90.1 ± 15 kg, 31.6 ± 6.6 kg/m^2^, 36.9 ± 7% fat) took part in a randomized, double-blind, and placebo-controlled study. The participants underwent baseline assessments and were then assigned to ingest 3 g/day of a placebo (PLA) or Momstamin 2′-F while initiating the exercise and weight-loss program. Follow-up tests were performed after 6 and 12 weeks. Data were analyzed using general linear model statistics with repeated measures and mean changes from baseline values with 95% confidence intervals (CIs). Results: No group × time × sex interaction effects were observed, so group × time effects are reported. Participants in both groups saw comparable reductions in weight. However, those with 2′-FL demonstrated a significantly greater reduction in the percentage of body fat and less loss of the fat-free mass. Additionally, there was evidence that 2′-FL supplementation promoted more favorable changes in resting fat oxidation, peak aerobic capacity, IL-4, and platelet aggregation, with some minimal effects on the fermentation of short-chain fatty acids and monosaccharides in fecal samples. Moreover, participants’ perceptions regarding some aspects of the functional capacity and ratings of the quality of life were improved, and the supplementation protocol was well tolerated, although a small, but significant, decrease in BMC was observed. Conclusions: The results support contentions that dietary supplementation of 2′-FL (3 g/d) can promote fat loss and improve exercise- and diet-related markers of health and fitness in overweight sedentary individuals initiating an exercise and weight-loss program. Further research is needed to explore the potential health benefits of 2′-FL supplementation in both healthy and elderly individuals (Registered clinical trial #NCT06547801).

## 1. Introduction

Human breast milk provides the optimal nutrition for newborns [1,2]. This is because human milk contains several bioactive compounds (e.g., carbohydrates, fats, proteins, and other molecules) that have been reported to promote health and immunity [3]. Infants fed breast milk have been reported to have a lower incidence of diarrhea, and children and adults fed breast milk as infants have been reported to have fewer food allergies, autoimmune diseases and less metabolic syndrome than infants fed formula [4,5,6]. Following lactose and lipids, HMOs are human milk’s most abundant solid component [7,8]. Because of their prebiotic and other health-related properties that affect immune cell functions, HMOs serve as an important immune defense system in human milk, which comprises a mixture of over 200 oligosaccharides [9]. Research has recently been focusing on the role of human milk oligosaccharides (HMOs) in health. 

Fucosyllactose (FL), which includes 2′-FL and 3-FL, represents the predominant category of human milk oligosaccharides (HMOs) in human milk, with 2′-FL comprising 20% of the total HMO content [10]. Animal studies have found that the oligosaccharide 2′-FL exhibits a variety of biological activities, such as leukocyte adhesion, interactions between the host and microbes, and neural development [1]. Moreover, 2′-FL is a primary prebiotic against infections and inflammation [11,12,13] and prevents weight gain while increasing thermogenesis and improving blood lipids [14]. Recent rodent studies have reported that supplementation of HMOs has antiplatelet and antithrombotic effects that reduce the size of ischemic brain injuries in mice [1] and reduce the rate of skeletal muscle atrophy [15]. In infants, metabolites from HMO degradation into short-chain fatty acids possess prebiotic effects by promoting the growth of desired bacteria, like *Bifidobacterium bifidum*, while inhibiting growth of undesired bacteria [16,17], thereby influencing bacteria–host interactions and the intestinal microbiota’s composition [11]. For this reason, HMOs have been added to infant formulae to promote the growth of bacteria that can promote health [11,18]. Adults supplemented with 2′-FL and lacto-N-neotetraose experienced an increase in *Bifidobacterium*, suggesting that HMOs may have prebiotic applications in nutrition that extend beyond infant formulae [19]. 

Recent studies have suggested that 2′-FL possesses anti-obesity properties by promoting fat oxidation through AMPK activation [20]. This activation upregulates genes involved in fatty-acid oxidation [21] and downregulates those related to lipid synthesis [22], facilitating a shift toward increased fat utilization. Additionally, 2′-FL may preserve fat-free mass by enhancing the integrity of the gut barrier [23], which aids in maintaining muscle mass during periods of weight loss or caloric restriction. Although energy restriction is traditionally advocated for obesity prevention, it can lead to a decreased REE because of reduced lean body mass [24,25], contributing approximately 20–30% to the total weight loss [26,27] and potentially stopping further weight loss. Consequently, preserving muscle mass is crucial for the success of weight-loss interventions. Theoretically, 2′-FL supplementation during exercise and dietary interventions may help to promote fat loss while maintaining muscle mass, reducing inflammation, and improving exercise-induced improvements in fitness and markers of health (e.g., blood lipids and glucose management). If so, participants may perceive improvements in the functional capacity and perceptions about the quality of life. 

This study assessed the impacts of 2′-FL supplementation on body composition, health, and fitness in overweight individuals initiating an exercise and energy-reduced dietary program. The primary outcomes were changes in bodyweight, body composition, and hip and waist circumferences. The secondary outcomes included physical activity assessment, diet, training adaptations, the resting energy expenditure, cell blood counts, blood lipids, glucose homeostasis, inflammatory markers, platelet aggregation, intestinal permeability, serum 2′FL levels, and fecal metabolome analysis. We hypothesized that 2′-FL supplementation would enhance training- and diet-induced changes in health and fitness markers. The following overviews the methods, results, and future research recommendations. 

## 2. Methods

### 2.1. Study’s Experimental Design

**Figure 1:**
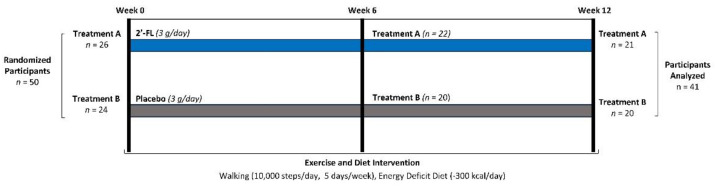
Study’s experimental design.

This research was conducted in a parallel-arm, randomized, double-blind, placebo-controlled fashion (see Figure 1). Approval was granted by the Human Protection Institutional Review Board (IRB2022-1559F, dated 4/25/23), adhering to ethical standards outlined in the Declaration of Helsinki for human participant research. The clinical trial was officially registered on clinicaltrials.gov (Trial ID: NCT06547801, registered on 8 September 2024). Dietary supplementation with milk oligosaccharides served as the independent variable. The primary outcomes included bodyweight, body composition, and hip and waist circumferences. The secondary outcomes included physical activity assessment, diet, training adaptations, the resting energy expenditure, cell blood counts, blood lipids, markers of glucose homeostasis, inflammatory markers, platelet aggregation, intestinal permeability, serum 2′FL levels, and fecal metabolome analysis. 

### 2.2. Study Participants 

Informed consent was obtained from all the subjects involved in this study. Healthy male and female participants were recruited for this study. The inclusion criteria were as follows: (1) men and women between 18 and 65 years; (2) the ability to adhere to the study’s protocols; (3) availability for the duration of the study, accommodating the timing of individual visits and scheduling needs; and (4) a body mass index (BMI) between 25 and 40 kg/ m^2^ and/or a percentage of body fat above 30%, preferably with a BMI of 25–32 kg/ m^2^, and a willingness to lose weight and engage in a fitness program. The exclusion criteria included (1) being pregnant, breastfeeding, or planning to become pregnant during the study period; (2) intending to make significant lifestyle changes (such as alterations in dietary or exercise habits or extensive travel) during the study; (3) engaging in regular exercise training or experiencing significant weight loss (>5%) within the last three months; (4) suffering from orthopedic issues that would inhibit participation in a general fitness program; (5) suffering from uncontrolled cardiovascular diseases, hypertension, diabetes, thyroid disorders, cancer, neurological conditions, or severe untreated mental health disorders that contraindicate physical activity; (6) using weight-loss supplements or medications within the previous four weeks; (7) having a history of alcohol or substance abuse in the past year; (8) currently a smoker (>1 pack/day in the last three months); and (9) having a known allergy to milk, lactose, or any related products.

**Figure 2:**
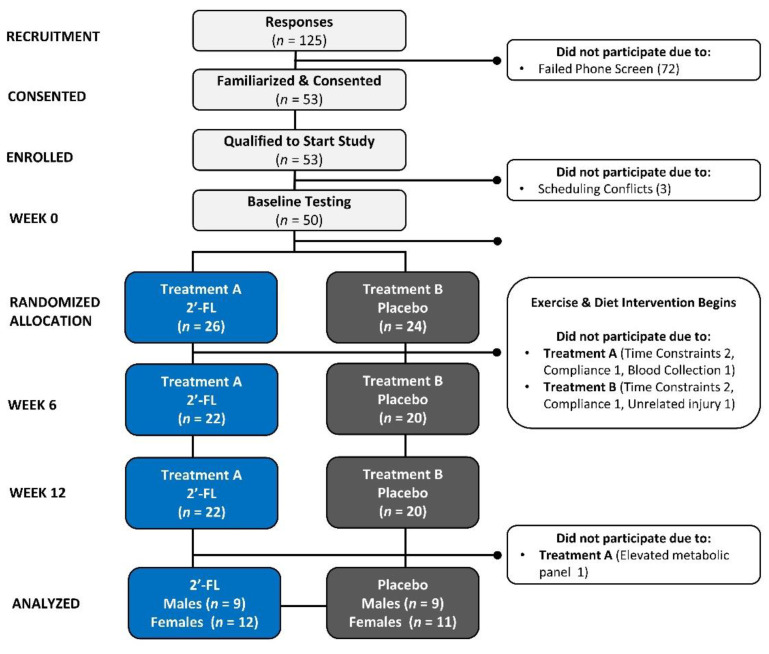
Consolidated Standards of Reporting Trials (CONSORT) diagram; 2′-FL represents 2′-fucosyllactose.

Figure 2 presents a Consolidated Standards of Reporting Trials (CONSORT) diagram. A total of 125 volunteers responded to advertisements and underwent pre-screening eligibility assessments. Out of these, 53 participants completed the phone screening, were acquainted with the study details, and gave informed voluntary consent to participate. A total of 50 participants completed the baseline testing, while 41 individuals (23 females and 18 males) completed the follow-up testing after 6 and 12 weeks of the intervention. Of those completing the study, 21 participants were in treatment group 1 (12 females and 9 males), and 20 (11 females and 9 males) were in treatment group 2. The reasons for participant withdrawal from the study included scheduling difficulties (4), inability to comply with the diet (2), difficulty in obtaining blood samples (1), injury unrelated to the study’s protocol (1), and an uncontrolled metabolic panel (1). 

### 2.3. Study Timeline

**Figure 3:**
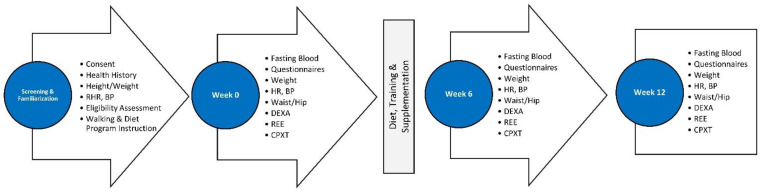
Testing progression. RHR is the resting heart rate, BP is the blood pressure, DEXA is a dual-energy x-ray absorptiometer, REE is the resting energy expenditure, and CPXT is the cardiopulmonary exercise test.

Figure 3 shows the study timeline and measurements made during participant visits. Recruitment strategies included e-mail campaigns, posts, and the distribution of flyers both online and in local print media. Potential volunteers first underwent a phone screening to evaluate their general suitability for the study. Those who met the initial phone criteria were scheduled for a familiarization session, where they received comprehensive information about the study and signed a voluntary consent to participate. Qualified volunteers were then randomized into the study and received instructions on documenting their diet. They were asked to document their fluid and food consumptions for four days before testing and fast for 12 h before lab visits. On the day of the testing, the participants brought their 4-day food diaries to the laboratory, provided fasting blood samples, and filled out questionnaires regarding side effects and the quality-of-life (QOL) assessment. The participants were then weighed and had body composition/bone density, resting heart rate, resting blood pressure, and anthropometric measurements determined. The participants then had the resting energy expenditure determined and performed a symptom-limited incremental maximal cardiopulmonary aerobic capacity test. Subsequently, the participants were randomly allocated to one of two supplementation groups. The participants were administered in a double-blind and randomized manner (matched according to sex and BMI) either 3 g/d of a placebo or 2′-FL. The participants participated in a 12-week walking program (5 days/week for 30 min/day, monitored using fitness apps) and a dietary intervention that involved reducing the energy intake by −500 kcals/d based on the resting energy expenditure and 4-day energy intake. The participants had weekly consultations with study personnel to monitor adherence and repeated all the testing at 6 and 12 weeks of the intervention. 

### 2.4. Dietary Intervention

The participants were instructed to adhere to a daily caloric intake of 1200, 1400, 1500, or 1600 kcal based on their resting energy expenditure. The diet was designed to promote a 300 kcal/day reduction in energy intake from the REE to subtract about a 500 kcal/day energy expenditure with increased physical activity. The dietary regimens adhered to the American Heart Association (AHA) guidelines for macronutrient distribution (i.e., 55% carbohydrate, 15% protein, and 30% fat). The participants received an exchange list that allowed for them to adjust their food choices to meet their specified caloric goals while adding variety to their meals. They were also provided with a target energy intake, a comprehensive weekly dietary plan, examples of meals, and options for food substitutions. To monitor their dietary intake, the participants used the MyFitnessPal application (MyFitnessPal Inc., Baltimore, MD, USA) [28]. Diet records were analyzed using Food Processor Nutrition Analysis version 11.14.9 software (ESHA Nutrition Research, Salem, OR, USA).

### 2.5. Training Intervention

The participants participated in a walking program (5 days per week for approximately 30 min per day) to accumulate approximately 10,000 steps daily. This was documented using exercise logs, or personal phones, activity monitors, and pedometers. 

### 2.6. Supplementation Protocol

Supplements were distributed to the participants in a double-blind and randomized fashion, employing a Latin square balanced design [29]. The experimental treatment included powdered Momstamin 2′-FL (Advanced Protein Technologies Corp., Gyeonggi-do, Republic of Korea), while the placebo consisted of powdered maltodextrin (Daesang Corp., Seoul, Republic of Korea). Both supplements were flavored with banana-flavored powder (LF04-1131, Samhwa F&F, Gyeonggi-do, Republic of Korea). The supplements had similar colors, tastes, and textures and were packaged in generically labeled foil stick packets for double-blind administration. The contents of the supplements were analyzed by the manufacturers for contents and purities. The participants were instructed to ingest the contents of one foil packet (3 g/day) in the morning with 8 ounces of water for 12 weeks. The dosage was determined based on the extrapolation of the dosages used in animal studies, to human dosing (i.e., 2–4 g/day), which improved strength, reduced platelet aggregation, and enhanced memory and cognitive functions. 

## 3. Procedures

### 3.1. Volunteer Demographics

Height and weight were obtained using a calibrated digital scale (Health-O-Meter Professional 500KL, Pelstar LLC, Alsip, IL, USA). Resting hemodynamic parameters were measured with participants seated, following a 5-minute rest period. Heart rates were assessed by palpating the radial artery, and resting blood pressures were measured through the auscultation of the brachial artery using a stethoscope and mercury sphygmomanometer according to established protocols [30]. Waist and hip circumferences were measured with a soft Gulick tape measure using standard procedures [31]. 

### 3.2. Body Composition

A Hologic Discovery W dual-energy X-ray absorptiometer (Hologic Inc., Waltham, MA, USA) equipped with APEX version 4.0.2 software (APEX Corporation Software, Pittsburgh, PA, USA) was used to determine the body compositions and bone mineral contents (BMCs) [32,33]. The coefficient of variation (CV) for performing day-to-day and inter-day scans in our lab has ranged from 0.31 to 0.45% for the BMC and total/fat-free masses, with a mean intraclass correlation coefficient of 0.98 [34].

### 3.3. Resting Bioenergetics

Resting bioenergetics were measured using a ParvoMedics TrueOne 2400 metabolic measurement system (ParvoMedics Inc, Sandy, UT, USA). This system was calibrated using a series 5530 three-liter syringe (Hans Rudolph Inc., Kansas City, MO, USA) prior to each testing session. During the resting bioenergetics study, the participants were positioned in the supine position with their knees and hips bent at a 90° angle on a cushion. They were instructed to relax yet remain awake for 20–30 min. After 10 min, the final five consecutive time points that exhibited less than 5% variance, corresponding to a fractional content of carbon dioxide (FeCO_2_) ranging from 1% to 1.2%, were selected to calculate average values [35,36]. Glucose and fatty-acid utilizations were estimated by calculating the respiratory quotient (exchange ratio) [37,38]. A CV of 5.3% with an interclass correlation of 0.92 has been documented in female athletes [39], while the manufacturer reports a CV of ±2% for a healthy population.

### 3.4. Aerobic Capacity Assessment

The symptom-limited maximal aerobic capacity was elicited on a TrackMaster 425 motorized treadmill (Newton, KS, USA) following the Bruce protocol [40]. A ParvoMedics TrueOne 2400 metabolic measurement system (ParvoMedics Inc, Sandy, UT, USA), with a TrueOne32 exercise system, measured the expired ventilation, oxygen, and carbon dioxide concentrations. The system calibration followed standard protocols using a series 5530 three-liter syringe (Hans Rudolph Inc., Kansas City, MO, USA). Electrocardiograms (ECG) were recorded via a Cardio-Card version 7.2 (Nassif Associates, Brewerton, NY, USA). At the same time, blood pressures were obtained by the auscultation of the brachial artery using a mercury sphygmomanometer, adhering to established guidelines. 

### 3.5. Blood Collection and Analysis

Fasting blood samples were obtained by certified phlebotomists using standard phlebotomy techniques [41]. Samples were collected in three serum separation tubes (SSTs) and one ethylenediaminetetraacetic acid (EDTA) tube (BD Vacutainer, Becton, Dickinson and Company, Franklin Lakes, NJ, USA). The SSTs sat at room temperature for approximately 15 min prior to centrifugation at 3000× *g* for 10 min in a cooled (4 °C) Thermo Scientific Heraeus MegaFuge 40R centrifuge (Thermo Electron North America LLC, West Palm Beach, FL, USA). Serum extracted from two of the SSTs was dispensed in microcentrifuge tubes (Eppendorf, Enfield, CT, USA) for use in platelet aggregation tests or was preserved at −80 °C for subsequent analysis. Platelet aggregation measurements were conducted using a Chrono-Log^®^ Model 592 whole-blood aggregometer (Havertown, PA, USA). The remaining tubes were sent to the Clinical Pathology Laboratory (Austin, TX, USA, CLIA #45D0505003, CAP Accreditation #21525-01) for analysis. This included a complete blood count with differentials on whole-blood samples and a comprehensive metabolic panel on serum samples and hemoglobin A1c. 

Alpco Diagnostics (Salem, NH, USA) enzyme-linked immunoassay (ELISA) kits were used to determine insulin levels using a BioTek Epoch 2 plate reader (BioTek Instruments, Winooski, VT, USA) equipped with BioTek Gen 5 software. The reported intra-assay Cv for this kit ranged from 3% to 10%, while the inter-assay CV ranged from 6% to 17%. The homeostatic insulin resistance, the glucose–insulin ratio, and the quantitative insulin sensitivity check (QUICKI) were calculated using mathematical models [42]. Serum cytokine levels were quantified using the Cytokine Human Magnetic 10-plex panel using a Luminex 200 instrument system coupled with a Milliplex analyzer (ThermoFisher Scientific, Vienna, Austria) and analyzed using version 4.3 xPONENTTM software. Inter-assay and intra-assay CVs ranged from 2% to 18% and from 3% to 10%, respectively. Frozen serum specimens were also sent to the study sponsor for blinded quantification of 2′-fucosyllactose using the methods outlined in Seydametova et al. [43]. 

### 3.6. Feces Collection and Analysis

Fecal samples were collected and stored at −80 °C until shipment on dry ice to collaborating labs. The intestinal permeability was examined at the University of Memphis under the direction of Marie van der Merwe, PhD, using standard procedures [44,45] with a zonulin stool ELISA kit (cat#30-ZONHU-E01, ALPCO, Salem, NH, USA). The “Easy Extraction device” (cat# 30-EZEX-100, ALPCO, Salem, NH, USA) was used to collect 15 mg of the sample by inserting the “notched” dipstick into the collected stool sample multiple times and then inserting it into the collection tube containing an extraction buffer. Stool samples were suspended by shaking the tubes until no samples remained in the notches. The zonulin concentration in the diluted sample was determined though a competitive procedure, following the manufacturer’s protocol. Briefly, the biotinylated zonulin-family peptide was introduced to 100 µL of diluted samples, standards, and controls. The samples were then incubated in microtiter plate wells with polyclonal antibodies against zonulin. Free target antigens in the samples compete for binding with biotinylated zonulin-family peptides to the polyclonal anti-zonulin-family peptide antibodies that are immobilized in the microtiter plate wells. Peroxidase-labeled streptavidin, which attaches to biotin, was introduced to each well of the microtiter plate, and peroxidase substrate tetramethylbenzidine (TMB) was introduced to induce a color change in the presence of the peroxidase. After the reaction was stopped, the absorbance was measured in a photometer at 450 nm. Using a standard curve, zonulin concentrations were calculated. 

Fecal metabolome analysis was performed at the University of California, Davis, under Dr. Carolyn M. Slupsky’s direction, using previously described methods [46,47,48,49]. Samples were manually homogenized in ice-cold Dulbecco’s phosphate-buffered saline, passed through a 0.22 µm pore-size syringe filter, and filtered using a 3 kDa ultra centrifugal filter. To assist in the quantification of the metabolites, an internal standard, DSS-d6 (trimethylsilyl-propane sulfonate) at a specified concentration, was added to the samples. Nuclear magnetic resonance (NMR) data were collected on a Bruker Avance 600 MHz NMR spectrometer, which features a Sample Jet autosampler (Bruker, BioSpin, Freemont, CA, USA). Measurements were conducted at 298 K utilizing the NOESY 1H pre-saturation experiment (‘noesypr1d’) with the parameters previously described [46]. Metabolites were measured based on the established concentration of the internal reference using Chenomx NMRSuite Profiler v.8.3 software [47]. The compounds listed in the database were verified by comparison to reference NMR spectra of pure compounds. This verification process is reproducible and accurate [48,49]. The reported concentrations were adjusted as previously described [46].

### 3.7. Physical Activity Status

Work, transportation, household, caregiving, and recreational physical activity levels were determined using the 7-day International Physical Activity Questionnaire (IPAQ). This questionnaire evaluates the frequency and intensity of physical activities within these categories based on established metabolic equivalent (MET) levels. The IPAQ is a reliable assessment of general changes in physical activity patterns [50]. 

### 3.8. Quality-of-Life Assessment

Subjective ratings of the quality of life (QOL) were evaluated using version 1 of the 36-item Short-Form Health Survey (SF-36) [51]. This survey provides a comprehensive evaluation of physical and mental health aspects, including physical functioning, which assesses engagement in vigorous activities without health constraints; role physical, assessing the capacity to manage work and everyday activities; bodily pain, which examines the extent to which pain hinders normal functioning; and general health, a self-evaluation of one’s health status. Additionally, it assesses vitality, capturing the sensation of energy and zest for life; social functioning, related to the ability to participate in usual social activities; role emotional, which look at how emotional problems affect daily activities and work; and mental health, which gauges the emotional state, including feelings of happiness, peace, and overall contentment. The SF-36v2 has a reported Cv ranging from 0.81 to 0.95 across all its domains [52,53]. 

### 3.9. Side-Effect Assessment

The frequency and severity of selected side effects were evaluated using separate Likert scales ranging from 0 (none, 1 = 1–2 occurrences per week) to 5 (very severe, 5 = ≥9 per week). CVs, in our lab, using this survey range from 1% to 3%, with intraclass correlations for single-item surveys between 0.6 and 0.88 [54,55,56].

### 3.10. Statistical Analysis

The sample size was calculated based on our prior research [56,57,58,59,60,61,62,63,64,65], with a 5% expected improvement in primary outcome variables and a statistical power of 80%. An n-size of 40 was deemed as sufficiently powered (20 per group) to assess clinically significant body composition changes. Data analysis was conducted utilizing IBM^®^ SPSS^®^ statistical software, version 29 (IBM Corp., Armonk, NY, USA). Mixed-model and general linear model (GLM) multivariate and univariate statistics, where the time (within-subject) factor was assessed using repeated-measures statistics and groups (between subjects), were evaluated as independent groups and were used to analyze the results. Sphericity was evaluated using Mauchly’s test, while normality was evaluated using skewness and kurtosis statistics. Alpha levels from Wilks’ lambda and Greenhouse–Geisser correction tests were used to evaluate time and group × time effects. Post hoc and pairwise comparisons were evaluated using Fisher’s least-significant difference test [66,67]. Partial eta-squared ($\eta_{p}^{2}$) values were employed to assess the effect size, with 0.01 representing a small effect, 0.06 a medium effect, and 0.14 a large effect size [68]. We also analyzed the data to assess whether sex differences influenced the results. Because no significant group × time × sex interaction effects were observed unrelated to body mass differences, we only report group × time effects unless otherwise specified. The data were considered as significant when the type I error was 0.05 or less, while statistical tendencies were identified when *p*-values ranged between 0.05 and 0.10 to inform the reader about potential effects and reduce the probability of type-II error CIs [69,70,71,72,73,74,75]. The clinical significance of the findings was evaluated based on mean changes accompanied by 95% confidence intervals (CIs). Changes were considered as clinically significant when the means and CIs were entirely above or below baseline values. Categorical questionnaires were analyzed using chi-squared analysis. 

## 4. Results

### 4.1. Volunteer Descriptive Data

Table S1 presents the participants’ descriptive data. The average age was 38.0 ± 12.9 years, with an average height of 170.7 ± 7.9 cm and a weight of 90.1 ± 15.4 kg. The participants had an average body mass index (BMI) of 31.6 ± 6.6 kg/m^2^, an average resting heart rate of 70.4 ± 12 bpm, an average resting systolic blood pressure of 119.0 ± 15 mmHg, an average resting diastolic blood pressure of 76.5 ± 8.5 mmHg, and an average peak aerobic capacity of 28.0 ± 5.9 mL/kg/min. No significant differences were seen between groups, except for the BMI, which was higher in the PLA group (*p* = 0.049). Sex differences were noted in all the baseline variables except for the age and resting heart rate. 

### 4.2. Physical Activity and Diet 

#### 4.2.1. Physical Activity

Table S2 presents the amount of walking that volunteers participated in during the study. Overall, the GLM analysis indicated no significant time effect (*p* = 0.116, $\eta_{p}^{2}$ = 0.107, moderate effect). However, there was a tendency for interaction between the groups (*p* = 0.091, $\eta_{p}^{2}$ = 0.119, moderate effect). Univariate analysis revealed that the 2′-FL group consistently achieved and maintained the 10,000-step-per-day goal, while the PLA group showed a significant reduction in the weekly step count by week 6. The pairwise comparison indicated that the 2′-FL group walked significantly more steps/week when assessed at weeks 6 and 12. 

**Figure 4:**
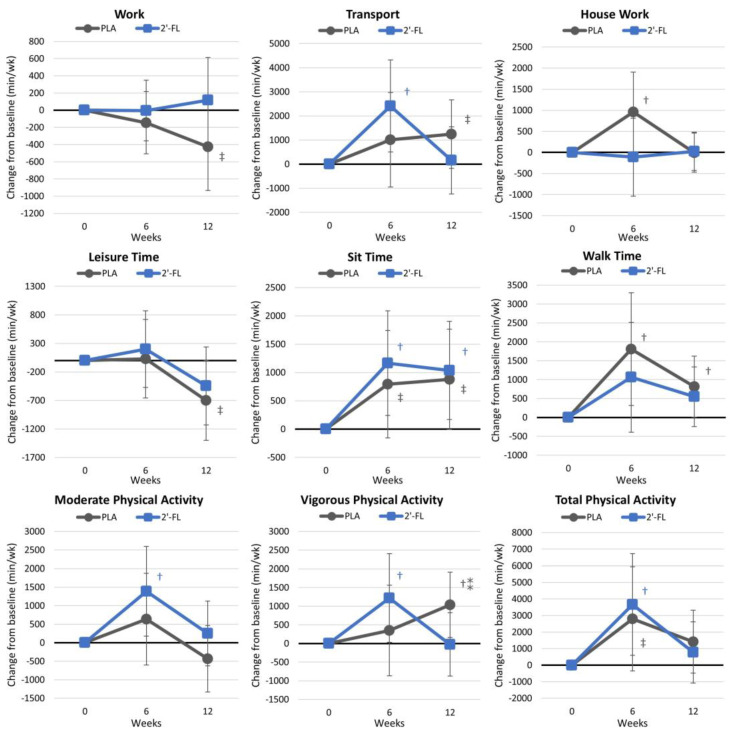
International Physical Activity Questionnaire results. Data are mean changes from baseline values with 95% confidence intervals. † = *p* < 0.05 (‡ = from *p* > 0.05 to *p* < 0.10) from baseline values. ⁑ = from *p* > 0.05 to *p* < 0.10) for differences between groups.

Table S3 presents results from the International Physical Activity Questionnaire. Overall, the GLM analysis showed a significant effect over time (*p* = 0.011, $\eta_{p}^{2}$ = 0.175, large effect), although no interaction effect between the group and time was observed (*p* = 0.147, $\eta_{p}^{2}$ = 0.122, moderate effect). Univariate analysis demonstrated significant time effects for minutes/week of transportation time, leisure time, sitting time, walking time, moderate physical activity time, and the total physical activity. Vigorous-physical-activity values tended to interact between treatments with increasing time effects after 6 weeks in the 2′-FL group and 12 weeks in the PLA group. Figure 4 shows the changes in IPAQ measures from baseline values. This analysis shows that the exercise intervention successfully increased physical activity in several areas in both treatment groups. 

#### 4.2.2. Energy and Macronutrient Intakes 

**Figure 5:**
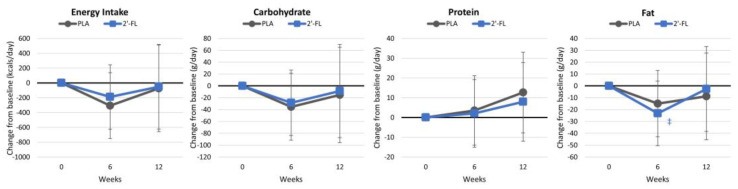
Energy and macronutrient intakes. Data are mean changes from baseline values with 95% confidence intervals. ‡ = from *p* > 0.05 to *p* < 0.10 from baseline values.

Table S4a displays the observed energy and macronutrient consumptions throughout the study period; while Table S4b shows fat-, fiber-, and carbohydrate-type intakes; and Table S4c displays micronutrient intakes. Overall, the GLM analysis showed no significant time (*p* = 0.131, $\eta_{p}^{2}$ = 0.078) or group × time (*p* = 0.625, $\eta_{p}^{2}$ = 0.040) effects. Univariate analysis indicated that the dietary intervention effectively reduced energy intakes after six weeks by about 246 kcal/day, as planned, although self-reported energy and macronutrient intakes were only 102 kcal/day below baseline values at 12 weeks. Figure 5 illustrates a decrease in fat consumption over time, with a tendency toward lower fat intake in the 2′-FL group at 6 weeks (−19.0 g [−38, 0.5], *p* = 0.056). No overall significant time (*p* = 0.297, $\eta_{p}^{2}$ = 0.198) or group × time (*p* = 0.702, $\eta_{p}^{2}$ = 0.153) effects were seen in the types of fat, fiber, and carbohydrate intakes or vitamin and mineral intakes (time *p* = 0.573, $\eta_{p}^{2}$ = 0.356; group × time (*p* = 0.349, $\eta_{p}^{2}$ = 0.387), although univariate analysis found a few time and between-group tendencies or differences. 

#### 4.2.3. Resting Energy Expenditure 

**Figure 6:**
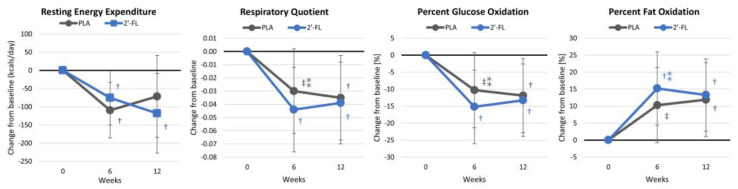
Energy expenditure data. Data are mean changes from baseline values with 95% confidence intervals. † = *p* < 0.05 (‡ = from *p* > 0.05 to *p* < 0.10) from baseline values. ⁑ = from *p* > 0.05 to *p* < 0.10) for differences between groups.

Weight loss typically reduces the resting energy expenditure, so a goal of effective weight loss is to prevent a large reduction in the REE. Table S5 shows the REE results. Overall, time effects were noted (*p* < 0.001, $\eta_{p}^{2}$ = 0.147, large effect), yet there were no significant differences between the treatment groups (*p* = 0.534, $\eta_{p}^{2}$ = 0.032, small effect). Univariate analysis revealed time effects for the resting energy expenditure (*p* = 0.009, $\eta_{p}^{2}$ = 0.127, large effect), respiratory quotient (*p* < 0.001, $\eta_{p}^{2}$ = 0.170, large effect), carbohydrate oxidation (*p* = 0.001, $\eta_{p}^{2}$ = 0.169, large effect), and fat oxidation (*p* = 0.001, $\eta_{p}^{2}$ = 0.169, large effect). However, no significant interactions were found in these variables. Figure 6 shows some effects, with fat oxidation increasing in the 2′-FL group after 6 weeks of supplementation compared to the PLA group. 

### 4.3. Primary Variables

**Figure 7:**
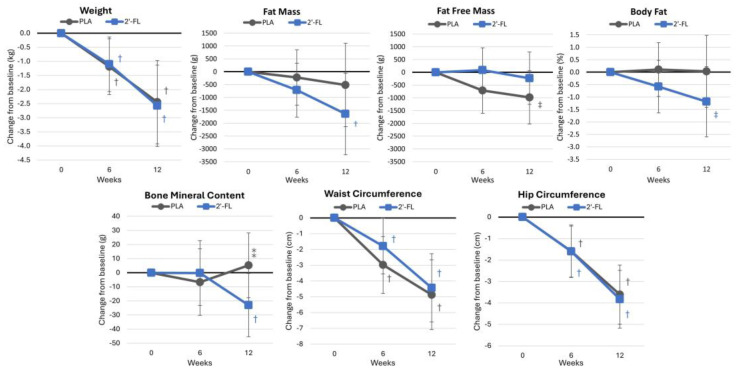
Bodyweight and body composition data. Data are mean changes from baseline values with 95% confidence intervals. † = *p* < 0.05 (‡ = from *p* > 0.05 to *p* < 0.10) from baseline values. ⁑ = from *p* > 0.05 to *p* < 0.10) for differences between groups.

Table S6 presents body composition and anthropometric result measurements, showing overall time effects (*p* < 0.001, $\eta_{p}^{2}$ = 0.417, large effect) without significant differences between the treatment groups (*p* = 0.760, $\eta_{p}^{2}$ = 0.065, moderate effect). Univariate analysis indicated time effects for bodyweight (*p* < 0.001, $\eta_{p}^{2}$ = 0.325, large effect), fat mass (*p* = 0.084, $\eta_{p}^{2}$ = 0.065, moderate effect), waist circumference (*p* < 0.001, $\eta_{p}^{2}$ = 0.361, large effect) and hip circumference (*p* < 0.001, $\eta_{p}^{2}$ = 0.500, large effect), with no significant time effects for the lean-tissue mass (*p* = 0.166, $\eta_{p}^{2}$ = 0.046, small effect), percentage of body fat (*p* = 0.368, $\eta_{p}^{2}$ = 0.025, small effect), or bone mineral content (*p* = 0.545, $\eta_{p}^{2}$ = 0.015, small effect). Hip circumference showed a tendency to vary between groups (*p* = 0.060, $\eta_{p}^{2}$ = 0.088, moderate effect), although no significant interaction effects were noted in the other body composition variables. Figure 7 indicates that there were no significant differences in bodyweight changes between the treatments at 6 and 12 weeks. However, following 12 weeks of the intervention, the 2′-FL group demonstrated clinically significant reductions in fat mass (−1642 g [−3226, −58], *p* = 0.043), in contrast to the PLA group, which showed only a modest decrease (−512 g [−2136, 1110], *p* = 0.527). Similar trends were seen in changes in the percentage of body fat (2′-FL: −1.19% [−2.50, 0.22], *p* = 0.096; PLA: 0.03% [−1.45, 1.48], *p* = 0.967). Additionally, there was evidence suggesting that the 2′-FL group was more successful in maintaining the fat-free mass (2′-FL: −229 g [−1250, 791], *p* = 0.652; PLA: −983 g [−2029, 63], *p* = 0.065). After 12 weeks, the BMC was significantly decreased below baseline values in the 2′-FL group (−22.9 g [−45, −0.5], *p* = 0.049) and tended to be lower than the PLA (−28.3 g [−60, 3], *p* = 0.082) unrelated to sex differences (group × time × sex *p* = 0.794, $\eta_{p}^{2}$ = 0.006, small effect). Meanwhile, no significant differences were found between treatments in waist or hip circumference measurements. 

### 4.4. Secondary Variables 

#### 4.4.1. Aerobic Capacity 

**Figure 8:**
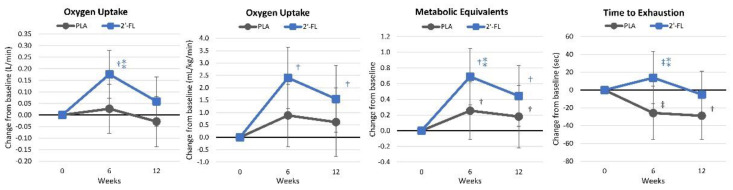
Changes in the aerobic capacity. Data are mean changes from baseline values with 95% confidence intervals. † = *p* < 0.05 (‡ = from *p* > 0.05 to *p* < 0.10) from baseline values. ⁑ = from *p* > 0.05 to *p* < 0.10 for differences between groups.

Table S7 presents the findings from the maximal aerobic capacity results. Although overall, time effects were noted (*p* < 0.001, $\eta_{p}^{2}$ = 0.257, large effect), there were no statistically significant differences observed between the treatment groups (*p* = 0.452, $\eta_{p}^{2}$ = 0.050, small effect). Univariate analysis identified time effects for the absolute peak aerobic capacity (*p* = 0.014, $\eta_{p}^{2}$ = 0.104, moderate effect), relative peak aerobic capacity (*p* = 0.001, $\eta_{p}^{2}$ = 0.159, large effect), and maximal metabolic equivalents (METS, *p* = 0.002, $\eta_{p}^{2}$ = 0.158, large effect), with no significant time effect for the time to exhaustion (*p* = 0.182, $\eta_{p}^{2}$ = 0.043, small effect). No significant interaction effects were observed between groups. Figure 8 reveals that changes from baseline values were greater after 6 weeks of the intervention and maintained to a higher degree with 2′-FL supplementation. 

#### 4.4.2. Cell Blood Counts

Table S8 presents the cell blood count results. Overall, the GLM analysis revealed no significant time (*p* < 0.650, $\eta_{p}^{2}$ = 0.158, large effect) or group × time effects (*p* < 0.657, $\eta_{p}^{2}$ = 0.158, large effect). Univariate analysis indicated that hematocrit (*p* = 0.079, $\eta_{p}^{2}$ = 0.064, moderate effect) and MCV values (*p* = 0.056, $\eta_{p}^{2}$ = 0.074, moderate effect) tended to change over time, though no significant differences were found in the other variables over time. Similarly, no significant treatment interactions were detected. 

#### 4.4.3. Blood Lipids

**Figure 9:**
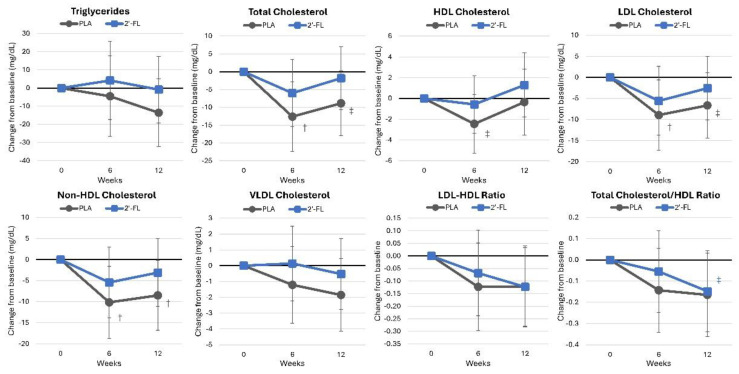
Changes in blood lipid levels. Data are mean changes from baseline values with 95% confidence intervals. † = *p* < 0.05 (‡ = from *p* > 0.05 to *p* < 0.10) from baseline values.

Table S9 presents the blood lipid data. Overall, the GLM analysis indicated no significant time effects (*p* < 0.247, $\eta_{p}^{2}$ = 0.094, moderate effect) or group × time interaction effects (*p* < 0.848, $\eta_{p}^{2}$ = 0.047, small effect). Univariate analysis revealed significant time effects for the total cholesterol (*p* = 0.014, $\eta_{p}^{2}$ = 0.107, moderate effect), LDL cholesterol (*p* = 0.026, $\eta_{p}^{2}$ = 0.094, moderate effect), and non-HDL cholesterol (*p* = 0.017, $\eta_{p}^{2}$ = 0.104, moderate effect), while changes in the LDL-to-HDL (*p* = 0.088, $\eta_{p}^{2}$ = 0.061, moderate effect) and total cholesterol-to-HDL (*p* = 0.074, $\eta_{p}^{2}$ = 0.064, moderate effect) ratios showed a tendency to change over time. No significant treatment interactions were noted. Mean changes from baseline values in blood lipids are shown in Figure 9. The PLA group exhibited small, yet more favorable, changes in blood lipid levels.

#### 4.4.4. Renal Function and Electrolytes

**Figure 10:**
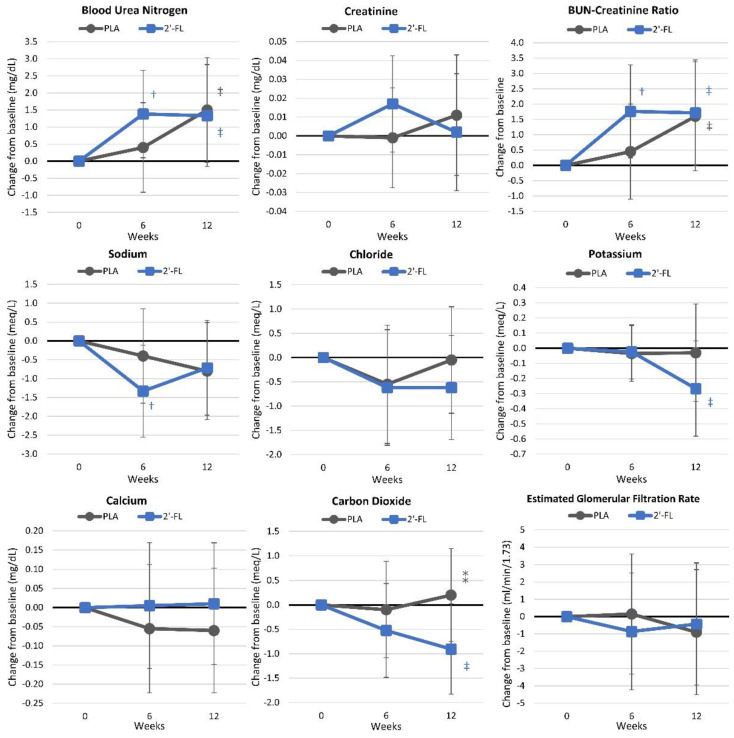
Changes in renal function and electrolytes. Data are mean changes from baseline values with 95% confidence intervals. † = *p* < 0.05 (‡ = from *p* > 0.05 to *p* < 0.10) from baseline values. ⁑ = from *p* > 0.05 to *p* < 0.10 for differences between groups.

Table S10 shows markers of renal function and electrolytes. Overall, the GLM analysis revealed no significant time effects (*p* < 0.524, $\eta_{p}^{2}$ = 0.109, moderate effect) or group × time interaction effects (*p* < 0.326, $\eta_{p}^{2}$ = 0.127, small effect). Univariate analysis showed significant time effects for BUN (*p* = 0.032, $\eta_{p}^{2}$ = 0.087, moderate effect) and the BUN-to-creatinine ratio (*p* = 0.029, $\eta_{p}^{2}$ = 0.089, moderate effect), while sodium (*p* = 0.087, $\eta_{p}^{2}$ = 0.061, moderate effect) exhibited a tendency to change over time. No significant treatment interactions were noted. Figure 10 shows the mean changes from baseline values, along with 95% confidence intervals. Some minor changes were observed over time within groups. However, only carbon dioxide levels showed a tendency to be lower in the group receiving the 2′-FL.

#### 4.4.5. Markers of Protein, Bone, and Liver Function

**Figure 11:**
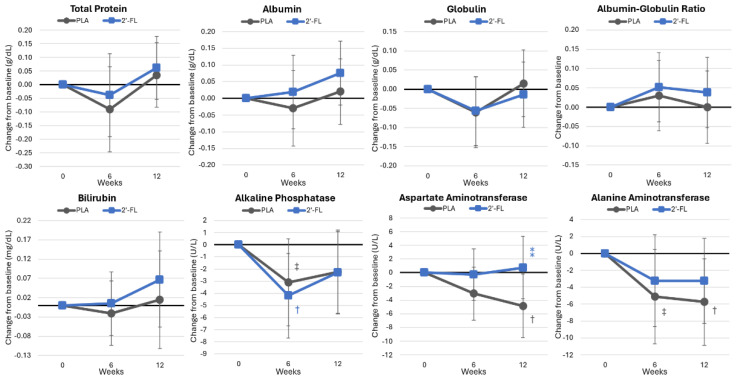
Changes in markers of liver function. Data are mean changes from baseline values with 95% confidence intervals. † = *p* < 0.05 (‡ = from *p* > 0.05 to *p* < 0.10) from baseline values. ⁑ = from *p* > 0.05 to *p* < 0.10 for differences between groups.

Table S11 presents protein and bone markers. Overall, the GLM analysis revealed time effects (*p* < 0.087, $\eta_{p}^{2}$ = 0.134, large effect) but no group × time interactions (*p* < 0.901, $\eta_{p}^{2}$ = 0.050, small effect). Univariate analysis indicated significant time effects for the total protein (*p* = 0.075, $\eta_{p}^{2}$ = 0.066, moderate effect), ALP (*p* = 0.033, $\eta_{p}^{2}$ = 0.088, moderate effect), and ALT (*p* = 0.021, $\eta_{p}^{2}$ = 0.100, moderate effect). No significant interactions between treatment groups and time were found. Aspartate aminotransferase and alanine aminotransferase levels tended to decrease in the PLA group (Figure 11). 

#### 4.4.6. Glucoregulatory Control

**Figure 12:**
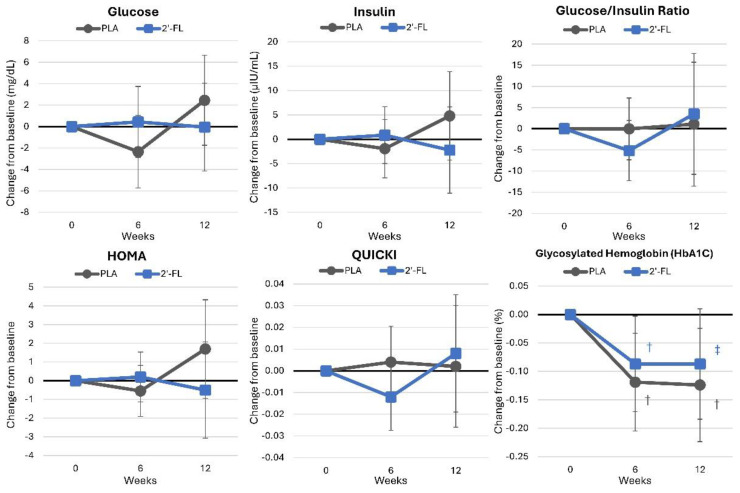
Changes in glucose and insulin sensitivity markers. Data are mean changes from baseline values with 95% confidence intervals. † = *p* < 0.05 (‡ = from *p* > 0.05 to *p* < 0.10) from baseline values.

Table S12 presents glucose, insulin, and HbA1c data. Overall, the GLM analysis revealed significant time effects (*p* = 0.028, $\eta_{p}^{2}$ = 0.141, large effect) but no interactions between group and time (*p* < 0.781, $\eta_{p}^{2}$ = 0.052, small effect). Univariate analysis indicated time effects for HbA1c. However, no statistically significant interaction effects between treatments were noted for glucose- and insulin-related markers. Changes from baseline values and 95% confidence intervals are presented in Figure 12. Time effects were evident in both groups, with no significant differences detected between the treatments. 

#### 4.4.7. Inflammatory Markers and Cytokines

**Figure 13:**
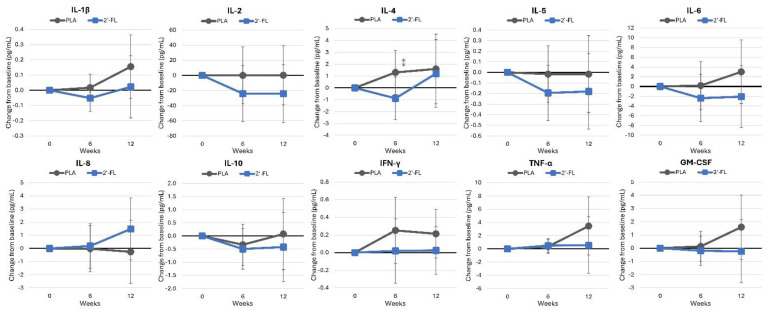
Changes in inflammatory cytokines. Data are mean changes from baseline values with 95% confidence intervals. ⁑ = from *p* > 0.05 to *p* < 0.10 for differences between groups.

Inflammatory markers and cytokines are shown in Table S13. Overall, the GLM analysis indicated no significant effects related to time (*p* = 0.798, $\eta_{p}^{2}$ = 0.095, large effect) or group-by-time interactions (*p* < 0.918, $\eta_{p}^{2}$ = 0.078, moderate effect) in IL-1β, 2, 4, 5, 6, 8, and 10; GM-CSF; INF-γ; or TNF-α. Likewise, no significant univariate time effects or group-by-time interactions were detected. Mean changes from baseline values with 95% CIs (see Figure 13) revealed lower values in the 2′-FL group. However, IL-4 levels were observed to be lower in the 2′-FL group after 6 weeks of supplementation, though this was only a tendency (−2.187 pg/mL [−4.8. 0.4], *p* = 0.095). 

#### 4.4.8. Platelet Aggregation

**Figure 14:**
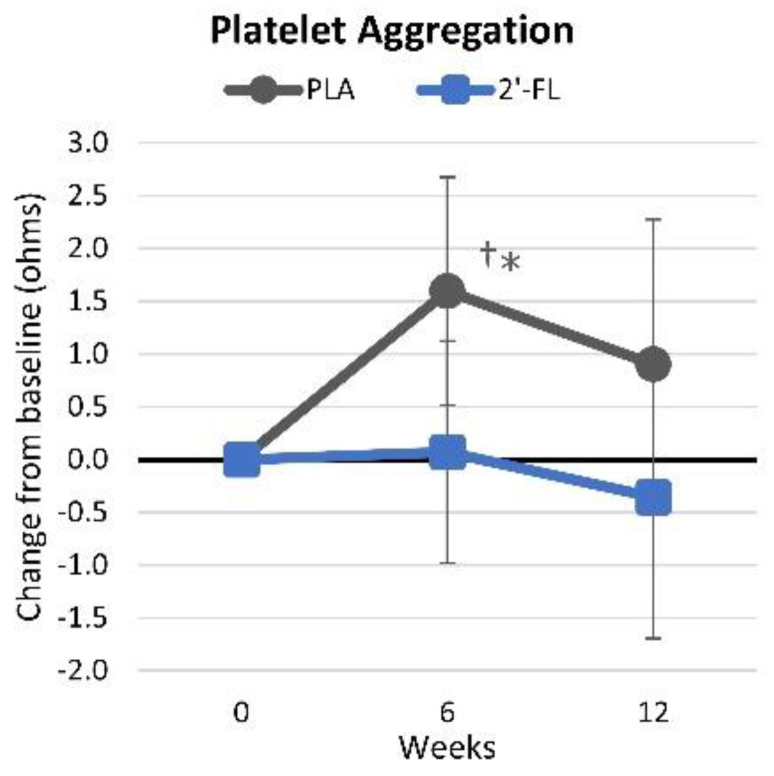
Changes in platelet aggregation impedance. Data are mean changes from baseline values with 95% confidence intervals. † = *p* < 0.05 from baseline values. * = *p* < 0.05 for differences between groups.

Table S14 presents platelet aggregation data. Increases in platelet aggregation values indicate greater impedance to flow. Multivariate analysis revealed time effects (*p* < 0.091, $\eta_{p}^{2}$ = 0.118, large effect) but no group-by-time interactions (*p* < 0.133, $\eta_{p}^{2}$ = 0.101, large effect) for platelet aggregation. Pairwise analysis showed significantly higher values in the PLA group compared to the 2′-FL group after 6 (2.30 ohms [0.65, 4.0), *p* = 0.008) and 12 weeks of the intervention (2.04 ohms [−0.09, 4.2), *p* = 0.06). The mean changes from baseline values are shown in Figure 14. Although the changes were small, the participants in the PLA treatment group observed slight increases in platelet aggregation values, whereas those in the 2′-FL group did not. 

#### 4.4.9. Intestinal Permeability

**Figure 15:**
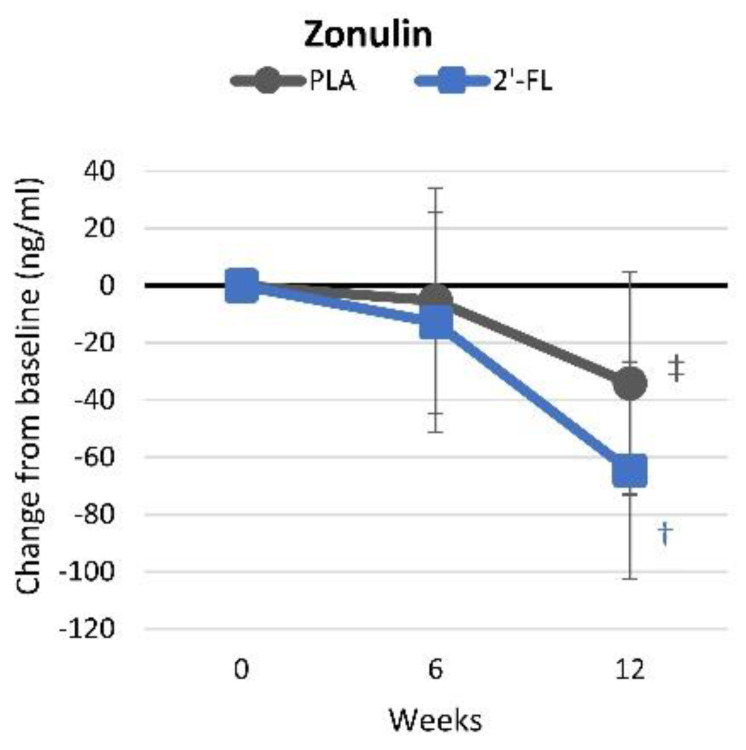
Changes in fecal zonulin intestinal permeability. Data are mean changes from baseline values with 95% confidence intervals. † = *p* < 0.05 (‡ = from *p* > 0.05 to *p* < 0.10) from baseline values.

Zonulin is an intestinal protein that is critical for epithelial tight junction formation. Elevated fecal zonulin levels are linked to impaired gut permeability and symptoms of leaky gut. Table S15 presents the zonulin intestinal permeability results. Multivariate analysis revealed significant time effects (*p* < 0.001, $\eta_{p}^{2}$ = 0.173, large effect) but no interactions (*p* = 0.476, $\eta_{p}^{2}$ = 0.038, small effect). Pairwise analysis indicated significant reductions in zonulin levels from baseline levels in the 2′-FL group, while in the PLA group, levels only tended to decrease. Notably, baseline zonulin levels were higher in the 2′-FL group, yet by the end of the study, levels were comparable between the groups. These changes are shown in Figure 15. 

#### 4.4.10. Serum 2′-Fucosyllactose Levels

**Figure 16:**
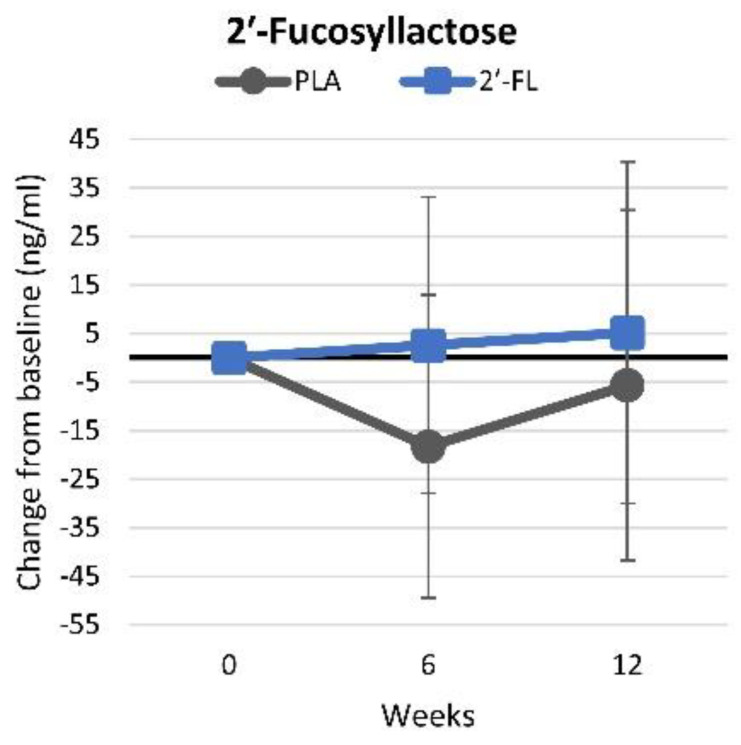
Changes in serum 2′-FL levels. Data are mean changes from baseline values with 95% confidence intervals.

Table S16 presents serum 2′-FL levels observed in fasting blood samples. Multivariate analysis did not reveal any statistically significant effects (*p* = 0.715, $\eta_{p}^{2}$ = 0.018) or interactions between the time and group (*p* = 0.636, $\eta_{p}^{2}$ = 0.024). However, pairwise analysis indicated reductions in 2′-FL levels in the PLA group following six weeks of supplementation, in contrast to the stable levels observed in the 2′-FL group. These trends were similarly evident when analyzing mean changes from baseline values with 95% CIs, although the difference between the treatment groups was no longer seen (Figure 16).

#### 4.4.11. Metabolome Analysis

**Figure 17:**
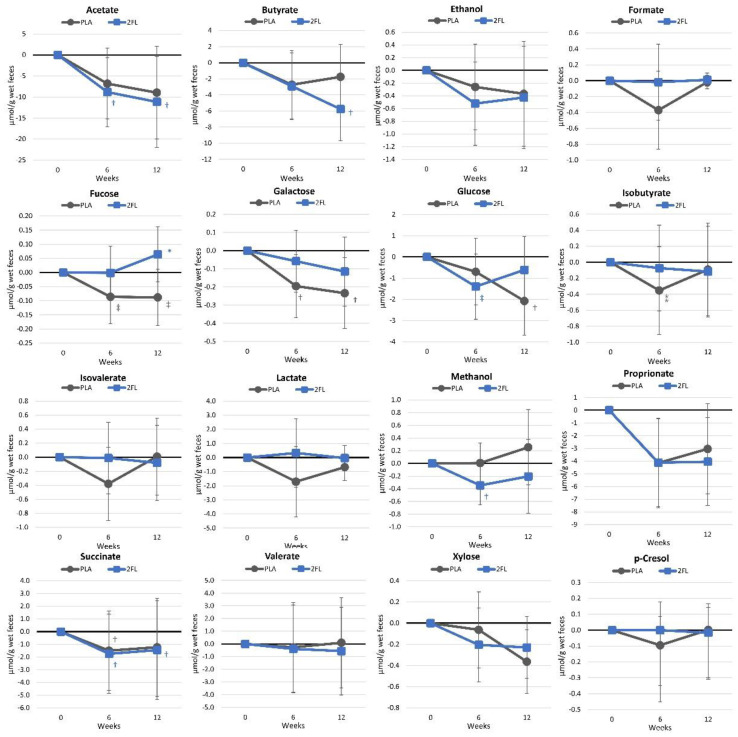
Changes in fecal metabolome metabolites. Data are mean changes from baseline values with 95% confidence intervals. † = *p* < 0.05 (‡ = from *p* > 0.05 to *p* < 0.10) from baseline values. * = 0/0–5 (⁑ = from *p* > 0.05 to *p* < 0.10) for differences between groups.

Table S17 shows the fecal metabolome analysis results. This analysis targeted fecal metabolites associated with the fermentation of monosaccharides, complex carbohydrates, and short-chain fatty acids (SCFAs). No 2′-FL, 3′-galactosyl-lactose, or lactose was present at quantifiable levels. 1,2-Propanediol, a byproduct of microbial 2′-fucosyllactose utilization, was only detectable in six samples. Therefore, these data were not analyzed. The GLM analysis of 17 metabolite markers revealed a statistical tendency time effect (*p* = 0.092, $\eta_{p}^{2}$ = 0.278, large effect), with no group × time interaction (*p* = 0.166, $\eta_{p}^{2}$ = 0.260, large effect). Univariate analysis found time effects for acetate (*p* = 0.024), butyrate (*p* = 0.024), galactose (*p* = 0.031), glucose (*p* = 0.038), propionate (*p* = 0.003), and xylose (*p* = 0.032). However, only the fucose values showed a tendency for interaction (*p* = 0.078, $\eta_{p}^{2}$ = 0.064, moderate effect). Mean changes from baseline values are presented in Figure 17. Acetate (weeks 6 and 12), butyrate (week 12), and methanol (week 6) decreased from baseline values with 2′-FL ingestion, while fucose (weeks 6 and 12), galactose (weeks 6 and 12), and glucose (week 12) significantly decreased with PLA ingestion. Week-12 fucose values were significantly higher with 2′-FL, while glucose values displayed a tendency to decrease by week 6. 

### 4.5. Quality of Life

Table S18 presents the QOL responses. The chi-squared analysis identified significant differences between groups in the following quality-of-life questions: Does health limit vigorous activity? Does health limit moderate activity? Does health limit the ability to climb several flights of stairs? Does health restrict bending, kneeling, or stooping? Does health limit the ability to walk more than a mile? Does health impede the ability to walk several hundred yards? Additionally, significant differences were observed between groups regarding how physical health affected reducing time spent on work or other activities, achieving less than desired; how emotional health influenced a reduction in time spent on work or other activities; aspects related to having ample energy versus feeling tired; and expectations concerning health deterioration. 

### 4.6. Health and Safety Assessments

#### 4.6.1. Resting Hemodynamics

**Figure 18:**
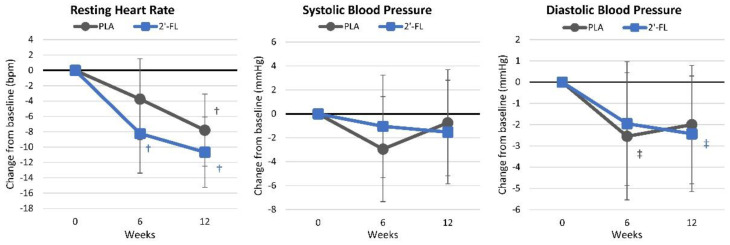
Resting hemodynamic results. Data are mean changes from baseline values with 95% confidence intervals. † = *p* < 0.05 (‡ = from *p* > 0.05 to *p* < 0.10) from baseline values.

Table S19 presents resting hemodynamic changes, and Figure 18 illustrates the mean changes from baseline values. Overall, the GLM analysis showed significant time effects (*p* < 0.001, $\eta_{p}^{2}$ = 0.179) but no group-by-time interactions (*p* = 0.774, $\eta_{p}^{2}$ = 0.021). Pairwise analysis indicated that the participants in the 2′-FL group exhibited a reduction in the resting heart rate from baseline values after six weeks, while a significant reduction in the PLA group was only observed after 12 weeks. No significant interaction effects were detected in the responses of the resting blood pressure. 

#### 4.6.2. Side-Effect Assessments

Table S20 shows the side-effect results. No significant differences were noted between groups regarding the frequency or severity of gastrointestinal distress, constipation, diarrhea, fatigue, abdominal discomfort, headache, heartburn, itching, facial swelling, skin rash, nasal congestion, or shortness of breath. These findings indicate that the dietary and exercise interventions were well tolerated. 

## 5. Discussion

This study evaluated the impacts of 2′-FL supplementation (3 g/day for 12 weeks) in conjunction with a walking intervention and an energy-reduced diet on weight loss, muscle mass preservation, the resting energy expenditure (REE), the aerobic capacity, and various health-related markers in sedentary and overweight individuals. The main findings suggest that 2′-FL supplementation induced more promising changes in body composition, enhanced the REE, increased the peak aerobic capacity, decreased IL-4, reduced platelet aggregation, promoted minor improvements in several fecal metabolites, improved intestinal permeability, and improved some perceptions about the QOL and functional capacity. Additionally, although the BMC tended to be lower in the 2′-FL group after 12 weeks of supplementation, 2′-FL supplementation was well tolerated and did not significantly affect whole-blood-cell counts, renal function and electrolytes, markers of protein, bone, and liver health, as well as resting hemodynamics. No group × time × sex differences were observed, suggesting that this nutritional intervention may be effective for both women and men. These findings indicate that 2′-FL supplementation may offer additional benefits to overweight individuals initiating an exercise and weight-loss program. The following provides a more detailed discussion of the results observed. 

### 5.1. Primary Outcomes

Recent studies have suggested that 2′-FL possesses anti-obesity properties by promoting fat oxidation through AMPK activation [20]. This activation upregulates genes involved in fatty-acid oxidation [21] and downregulates those related to lipid synthesis [22], facilitating a shift toward increased fat utilization. Additionally, 2′-FL may preserve fat-free mass by enhancing the integrity of the gut barrier [23], which aids in maintaining muscle mass during periods of weight loss or caloric restriction. Although energy restriction is traditionally advocated for obesity prevention, it can lead to a decreased REE because of reduced lean body mass [24,25], contributing approximately 20–30% to the total weight loss [26,27] and potentially stopping further weight loss. Consequently, preserving muscle mass is crucial for the success of weight-loss interventions. 

In our experiment, both the PLA and 2′-FL groups experienced similar weight losses over a 12-week weight-loss intervention. However, the 2′-FL group preserved muscle mass during weight loss, while the PLA group lost muscle mass. Although the differences between the groups were not statistically different, these findings suggest that 2′-FL supplementation may aid in preserving muscle mass during a weight-loss intervention. Furthermore, although the reduction in the body fat mass between the groups was not statistically significant, the 2′-FL group exhibited a clinically more significant decrease in body fat. It demonstrated a tendency toward greater changes in the percentage of body fat over time, suggesting more pronounced enhancements in body composition. 

Consistent with our results, Li et al. [14] investigated whether 2′-FL can mitigate weight gain resulting from a high-fat diet. The researchers assigned five-week-old male C57BL/6J mice to three groups: (1) a normal diet group (70% carbohydrates, 20% protein, and 10% fat); (2) a high-fat diet group (20% carbohydrates, 20% protein, and 60% fat); and (3) a 2′-FL supplementation group, which received a high-fat diet supplemented with 2′-FL (400 mg/kg). After 8 weeks, they reported that the mice in the 2′-FL supplementation group had a statistically significant reduction in bodyweight gain compared to those on a high-fat diet alone. This effect was attributed to increased uncoupling protein 1 (UCP1) expression. Additionally, 2′-FL supplementation has been linked to an increase in secondary bile acids [76], which are known to activate nuclear receptors that enhance the UCP1 pathway in both brown and white adipose tissues. This activation promotes thermogenesis and assists in mitigating obesity resistance caused by high-calorie diets [77]. Similarly, Lee et al. [78] observed that mice consuming a high-fat diet supplemented with 10% FL experienced significantly less weight gain compared to those consuming a high-fat diet without supplementation. Paone et al. [79] reported that 2′-FL supplementation significantly suppressed bodyweight gain in mice on a high-fat diet. Additionally, Gart et al. [80] found decreased lipid deposition in the livers of male Ldlr-/-.Leiden mice, thereby significantly lowering liver steatosis. Moreover, 2′-FL supplementation resulted in a significant reduction in bodyweight and fat-mass gain in mice on a high-fat diet [23,79]. Although these experiments were carried out in mice, they offer a theoretical rationale for how 2′-FL supplementation could influence fat oxidation and accumulation and fat-free mass preservation and potentially contribute to weight reduction.

Despite these favorable changes in body composition, 2′-FL supplementation was linked to a reduction in bone mineral content, though the difference between groups was only 28 g. Although the exact mechanism remains unclear, continuous energy restriction leads to significantly greater bone loss compared to that in individuals who maintain their regular diet [81,82]. Thus, further research is warranted to determine whether this outcome is an anomaly or if there are any synergistic adverse effects on bone mineral content with 2′-FL supplementation during energy restriction. Given the current findings, incorporating a 2′-FL supplement into a weight-loss program could offer additional benefits for weight management during periods of weight loss. 

### 5.2. Secondary Outcomes

The participants in the 2′-FL group demonstrated significant increases in peak oxygen uptake (ml/kg/min) at both week 6 and week 12 compared to baseline values. Although these markers initially improved, slight declines in aerobic capacity indicators were noted by week 12. This trend suggests an initial rise in fat oxidation (15.21%) at week 6 with a subsequent decrease by week 12, which helps in conserving muscle glycogen, potentially enhancing endurance performance [83]. The increase in the aerobic capacity observed after 12 weeks of supplementation with 2′-FL can also be explained by enhanced AMPK activity, which promotes PGC-1α activity, a key factor in skeletal muscle angiogenesis [84] and mitochondrial biogenesis [85]. Enhanced mitochondrial activity and function optimize the effectiveness of aerobic energy generation, leading to improved fat utilization during exercise [61].

Despite limited research on the effects of 2′-FL supplementation on platelet aggregation without abnormal bleeding, one study found supplementation had potential antiplatelet effects in mice without causing bleeding. These effects were attributed to the modulation of the GPVI-mediated signalosome, which subsequently affects granule secretion, thromboxane B2 production, serotonin release, and calcium mobilization. Consistent with prior reports, we found that the PLA group exhibited an increase in platelet aggregation compared to that in the 2′-FL group after 6 weeks of the intervention (2.30 ohms [0.65, 4.0], *p* = 0.008) and showed a trend toward higher aggregation after 12 weeks (2.04 ohms [−0.09, 4.2], *p* = 0.06). Further research is needed to fully evaluate 2′-FL’s effects on platelet aggregation.

We found some evidence that 2′-FL supplementation significantly improved intestinal permeability. Fecal zonulin levels were significantly reduced in the 2′-FL group. It is important to note that although baseline zonulin levels were initially greater in the 2′-FL group, by the end of the study, they were similar to those in the PLA group, suggesting the effectiveness of 2′-FL supplementation in decreasing intestinal permeability. Our findings support those of Kim et al. [86], who reported that reductions in zonula occludens-1 and occludin protein expressions as well as increased claudin-2 expression, induced by dextran sodium sulfate treatment in mice, were reversed by 2′-FL supplementation, indicating improved tight junction function and intestinal integrity. Additionally, 2′-FL was found to have the most significant protective effect on the gut epithelial barrier’s integrity among various HMO components [87] and was identified as the most potent HMO in preventing rises in epithelial permeability caused by inflammation [88].

Regarding fecal metabolites, the current study observed significant reductions in acetate, butyrate, and propionate, along with significant increases in fucose levels in the 2′-FL group. Reduced fecal SCFA levels in feces suggest changes in microbial composition and may also indicate greater absorption/utilization. For example, De la Cuesta-Zuluaga et al. [89] suggested that elevated levels of SCFAs in fecal samples could indicate a decreased absorption rate of SCFAs. Additionally, the authors have indicated that overweight individuals exhibit higher fecal SCFA levels compared to their lean counterparts, which are associated with increased gut permeability and dysbiosis [89,90]. These findings highlight the potential of 2′-FL as a prebiotic that enhances the body’s absorption and utilization of SCFAs. The increase in the fecal fucose content suggests that the 2′-FL consumed was likely fermented by gut bacteria by cleaving and releasing fucose and, thus, may impact the gut microbial composition. A longer supplementation period with a higher dosage of 2′-FL may produce greater differences.

The current study also examined the impacts of 2′-FL on resting lipid profiles, insulin responses, inflammatory markers, and subjective perceptions of the QOL. Although previous research has highlighted the potential impacts of 2′-FL on these parameters [14,80,87,91], our findings indicate that supplementation with 2′-FL did not significantly alter blood lipid profiles, except for improvements in HDL levels. Moreover, although there were tendencies toward improvement in all the glucose- and insulin-related markers by week 12, these changes were not statistically significant. Similarly, no significant differences were observed in pro-inflammatory cytokines at weeks 6 and 12; however, the 2′-FL group experienced less inflammation compared to the placebo (PLA) group throughout the study. Importantly, unlike most animal studies that induce elevated inflammation levels before administering 2′-FL, the subjects in our study maintained inflammatory cytokine levels within normal reference ranges, and clinical health-related blood parameters also remained within normal ranges. The current study also noted significant improvements in the subjective perceptions of both vigorous and moderate activities following 2′-FL supplementation. The participants reported enhanced abilities to climb several flights of stairs, bend, kneel, stoop, and walk, coupled with decreased perceptions of health deterioration. Because perceived limitations in physical activity and exercise can discourage individuals from engaging in weight-loss programs [92], improved perceptions of the quality of life through 2′-FL supplementation may promote greater adherence to these programs. Lastly, other than a small reduction in the BMC from baseline values with 2′-FL supplementation, which tended to be lower than PLA values at week 12 with no group × time × sex differences, 2′-FL supplementation was well tolerated, with no adverse side effects reported.

### 5.3. Limitations and Future Directions

Although these results are promising, several limitations should be considered. First, the exercise intervention was limited to walking and using steps/day to assess compliance. Although this exercise intervention is standard for overweight individuals starting a weight-loss program, additional benefits might have been observed if the participants had engaged in higher-intensity and supervised training incorporating a resistance exercise program. Second, this study evaluated a single dose (3 g/day) ingested in the morning of 2′-FL for 12 weeks. A higher dose, ingesting supplements before each meal, and/or supplementation for a longer period may promote greater benefits. Third, blood samples were obtained after a 12-hour fast, which may have minimized the effects of the 2′-FL supplementation on blood levels. Fourth, although we conducted a comprehensive study, we did not assess oxidative stress markers, fecal microbiota, or gut microflora assays because of funding limitations. Consequently, additional research may be prudent to (1) conduct additional pharmacokinetic studies to establish a dose–response relationship and the length of time that 2′-FL is present in the blood; (2) conduct studies involving more frequent and higher doses over an extended period; (3) evaluating the effects of 2′-FL in conjunction with supervised training that incorporates a resistance exercise program; (4) exploring the effects of 2′-FL ingestion before exercise on carbohydrate and fat oxidations during exercise and training in trained participants; (5) examining the effects of 2′-FL in older individuals to determine if it may improve the physical capacity and quality of life; (6) assessing the impacts of 2′-FL on additional health markers (e.g., oxidative stress markers and cognitive functions); and, (7) although expensive, conducting additional microbiome, gut microflora, and metagenomic analyses on a larger cohort.

## 6. Conclusions

The results support contentions that dietary supplementation of 2′-FL (3 g/d) can promote additional fat loss, aerobic training adaptations, improvements in some markers of health and inflammation, and perceptions about the quality of life, particularly those related to the physical capacity. Dietary supplementation of 2′-FL also reduced intestinal permeability and platelet aggregation, which may have clinical implications. There was also evidence that weight loss, and possibly 2′-FL, impacted the gut microbiome. No group × time × sex differences were observed, suggesting this nutritional intervention may be beneficial for both women and men. The supplement was well tolerated, although those in the 2′FL group had a small reduction in BMC that tended to be lower than PLA values at week 12, unrelated to sex differences. Further research is needed to assess the potential health benefits of 2′-FL supplementation in healthy and older individuals, its mechanism of action, and whether the change in BMC observed was an anomaly or a potential concern.

## Data Availability

Data and statistical analyses are available for non-commercial scientific inquiry and/or educational use upon request to the corresponding author as long as the use of the data does not violate IRB restrictions and sponsored research agreements and the authors and sponsors of this work are appropriately acknowledged.

## Supplementary Materials

The following supplemental materials are available online at https://www.mdpi.com/article/10.3390/nu16193387/s1: Table S1: Participant demographics; Table S2: Walking steps; Table S3a: International Physical Activity Questionnaire results; Table S3b: IPAQ physical activity scores; Table S4a: Energy and macronutrient intakes; Table S4b: Carbohydrate-, fiber-, and fat-type analysis data; Table S4c: Micronutrient analysis; Table S5: Resting energy expenditure; Table S6: Body composition measures; Table S7: Aerobic capacity measures; Table S8: Complete blood count results; Table S9: Serum blood lipid results; Table S10: Serum renal function markers; Table S11: Serum liver function markers; Table S12: Markers of insulin sensitivity; Table S13: Inflammatory marker and cytokine results; Table S14: Platelet aggregation results; Table S15: Fecal zonulin intestinal permeability results; Table S16: Serum 2′-fucosyllactose levels; Table S17: Fecal metabolome metabolite analysis data; Table S18: SF-36 quality-of-life results; Table S19: Resting hemodynamics; Table S20: Frequency and severity of side effects.
